# Giant isolated hydatid lung cyst: two case reports 

**DOI:** 10.1186/s13256-020-02524-4

**Published:** 2020-10-24

**Authors:** Jay Lodhia, Samwel Chugulu, Adnan Sadiq, David Msuya, Alex Mremi

**Affiliations:** 1 Department of General Surgery, Kilimanjaro Christian Medical Center, P.O. Box 3010, Moshi, Tanzania; 2 Kilimanjaro Christian Medical University College, P. O. Box 2240, Moshi, Tanzania; 3 Department of Radiology, Kilimanjaro Christian Medical Center, P.O. Box 3010, Moshi, Tanzania; 4 Department of Pathology, Kilimanjaro Christian Medical Center, P.O. Box 3010, Moshi, Tanzania

**Keywords:** Echinococcus, Hydatid cyst, Pulmonary, Tanzania, Zoonoses

## Abstract

**Background:**

Echinococcosis is a parasitic disease caused by *Echinococcus granulosus* and causes cystic lesions in the liver and lungs commonly. It is endemic in many parts of the world, and though humans are incidental hosts of the parasite, the disease can have severe consequences.

**Case presentation:**

We present two patients from pastoralist (Maasai) communities in rural Tanzania with long-standing chest pain accompanied by hemoptysis. Both were managed surgically after diagnosis, but one patient died of the complications following rapture of the cyst during surgery. Histopathological evaluation of the specimens confirmed the diagnosis of giant hydatid cysts.

**Conclusion:**

Animal-keeping communities such as the Maasai are at risk of echinococcosis because of their close proximity to animals. The diagnosis can be made on the basis of history and radiological as well as laboratory findings. Surgery is a recommended mode of treatment, though it carries a high risk, especially when the cyst ruptures. Primary preventive measures are thus necessary in order to avoid the secondary and tertiary complications of the management of giant hydatid cysts, which is difficult in resource-limited endemic areas.

## Background

Hydatid disease is a zoonotic parasitic infestation caused by tapeworms, particularly *Echinococcus granulosus* [1]. The life cycle of *Echinococcus* involves a definitive host and intermediate host [1]. Dogs (or other carnivores) harbor the adult tapeworms in their small intestine, and eggs are excreted into feces. The excreted ova, once ingested by the intermediate host (commonly sheep), develop into a hexacanth embryo in the liver through the portal circulation. Embryos in the liver develop cysts [2]. Humans are incidental intermediate hosts by ingestion of water or vegetables contaminated with echinococcal ova [2].

The treatment of hydatid lung cyst often requires surgical removal combined with chemotherapy (albendazole and/or mebendazole). The Maasai, a seminomadic indigenous group in northern Tanzania, are frequently in close contact with animals and therefore at higher risk of contracting the disease. Due to their culture and poor health-seeking behavior, such ethnic groups present late to medical facilities and opt for traditional remedies in the early stages, hence increasing their risk of poor treatment outcome. Disease awareness is therefore needed among the population and also health care workers for prevention and early management and to reduce complications.

## Case presentation

### Patient 1

A 64-year-old man, a Maasai from Ngorongoro District, Arusha Region, northern Tanzania, presented to our facility in February 2020 with chief complaints of chest pain and cough for the past 3 years. He further reported that, at times, he experienced a sore throat, blood-stained sputum, pain that was greater on the right side of the chest, and intermittent low-grade fevers. The patient also reported significant unintentional weight loss and generalized body weakness. He had been consuming local herbs with occasional relief and was unable to afford modern hospital care expenses. Despite using traditional medicines, he had no notable improvements. He had no significant past medical history, denied tuberculosis contact, and consumed local brew. He denied any formal education and was a farmer and livestock keeper. Upon examination, he was cachexic with mild conjunctival pallor and saturating at 97% on room air. His axillary temperature was 36.4 °C; his pulse rate was 95 beats per minute; and his blood pressure was 100/70 mmHg. His chest examination revealed reduced air entry on the right side. His abdomen was flat and moved with respiration with traditional marks on the upper quadrants, with no tenderness, and with a liver span of 10 cm. The finding of his neurologic examination was unremarkable. His hemoglobin level was 12.8 g/dl with an erythrocyte sedimentation rate (ESR) of 110 mm/hour. His creatinine level was 83 μmol/L, serum urea was 4.14 mmol/L, aspartate aminotransferase was 17.52 U/L, alanine aminotransferase was 23.78 U/L, and serum electrolytes were within normal range.

A CT scan of the patient’s thorax showed a massive cyst in the right lower lung measuring 19 × 11 cm with well-defined margins. A working diagnosis of bronchogenic cyst was reached with the possibility of hydatid cyst. Ultrasonography excluded the presence of the disease in the abdominopelvic region.

**Fig. 1:**
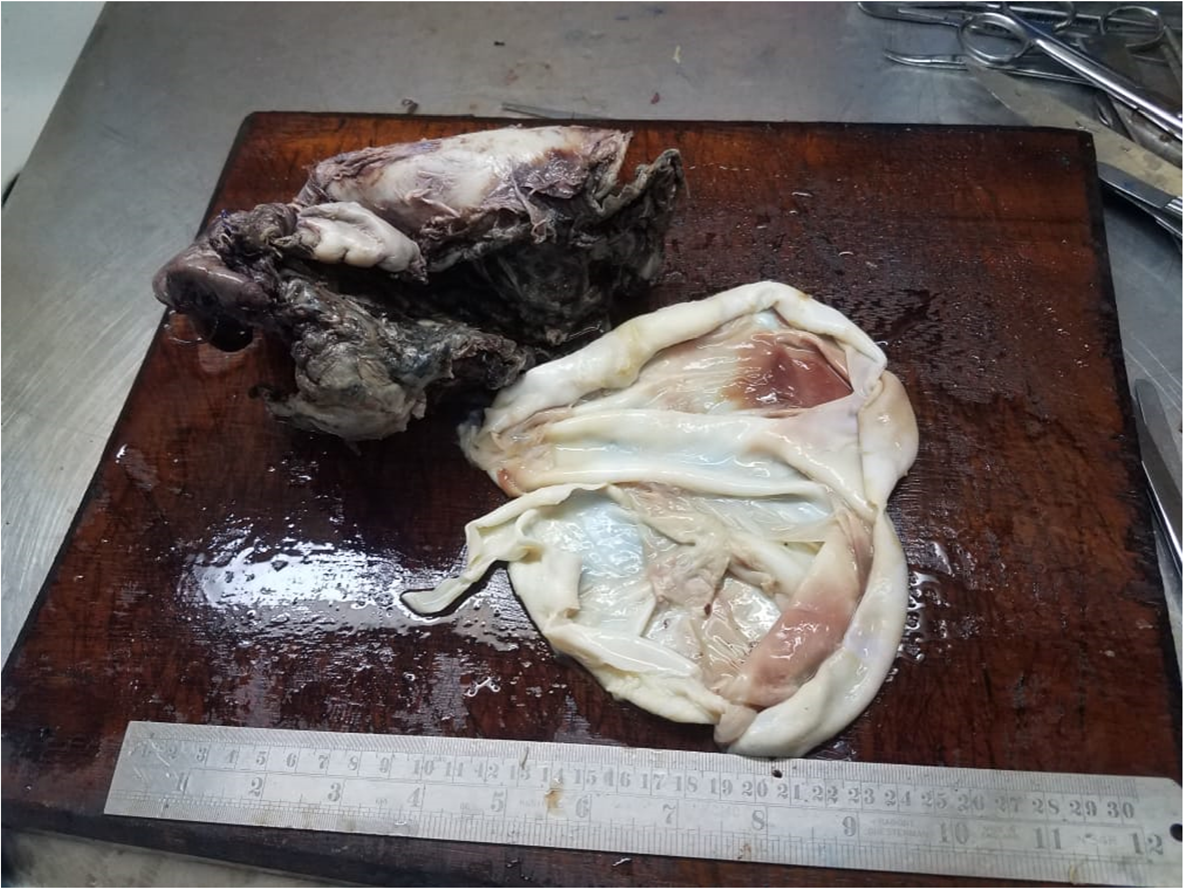
Ruptured cyst attached to lung parenchyma

**Fig. 2:**
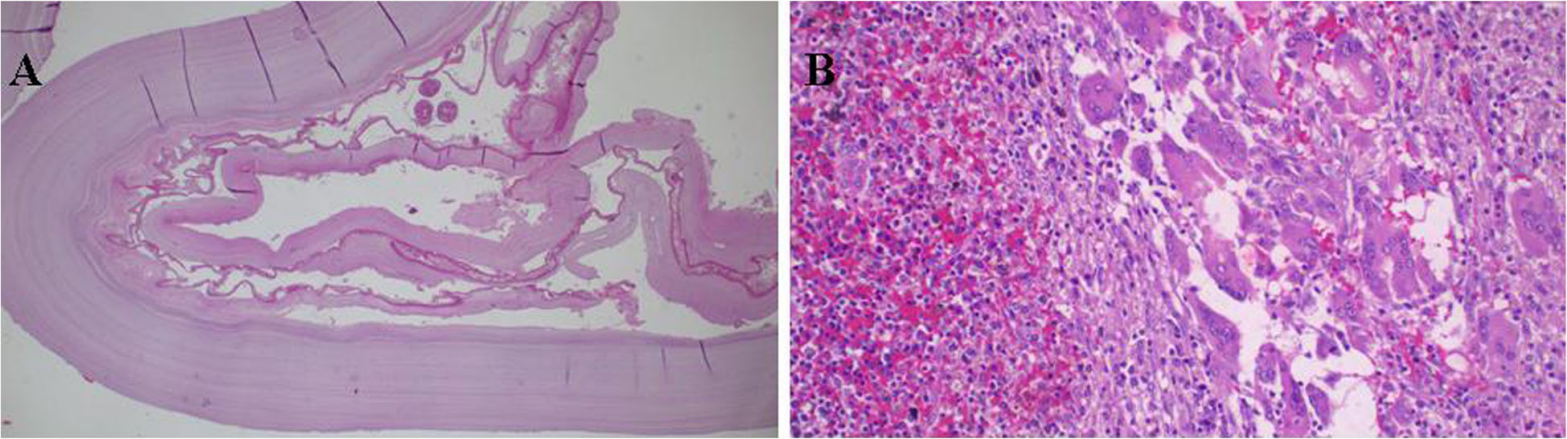
**a** Bilayered daughter cysts developing in large mother cysts and scolices. The worm produces sediment, so-called hydatid sand (Hematoxylin & eosin (H&E) staining; × 10 original magnification). **b** Lung sections with mixed chronic inflammation consisting mononuclear cells, eosinophils, and multinucleated giant cells. (H&E staining; × 20 original magnification)

We performed a right-sided sixth-interspace thoracotomy. Intraoperatively, we found a large hydatid cyst occupying the middle and lower lobes. During blunt dissection, the cyst ruptured, but the middle and lower lobes were successfully removed (Fig. 1). Approximately 30 minutes after surgery, the patient died in the intensive care unit (ICU) of sudden cardiac arrest. The probable cause of death we speculate to be anaphylaxis triggered by leakage from the ruptured cyst. The specimen taken intraoperatively was analyzed and confirmed to be a hydatid cyst of the lung (Fig. 2).

### Patient 2

A 13-year-old Maasai boy from rural Arusha, northern Tanzania, presented with a 2-month history of gradual and progressive dyspnea and cough that was dry initially but had started to produce blood-stained sputum in the past 2 weeks before admission. His past medical history was unremarkable, though his father reported that the child had lost significant weight through the course of the illness and had experienced intermittent low-grade fevers. The boy had no history of tuberculosis contact. The informant reported that before this admission, the boy had received treatment for his current illness from a local dispensary, which included antibiotics that he could not specify; however, the boy had no significant improvement. He is from a farming family and keeps livestock. Upon examination, the child was moderately pale, not cyanotic, and saturating at 98% on room air. His pulse rate was 85 beats per minute; his resting blood pressure was 108/60 mmHg; and he was afebrile with a temperature of 36.7 °C.

**Fig. 3:**
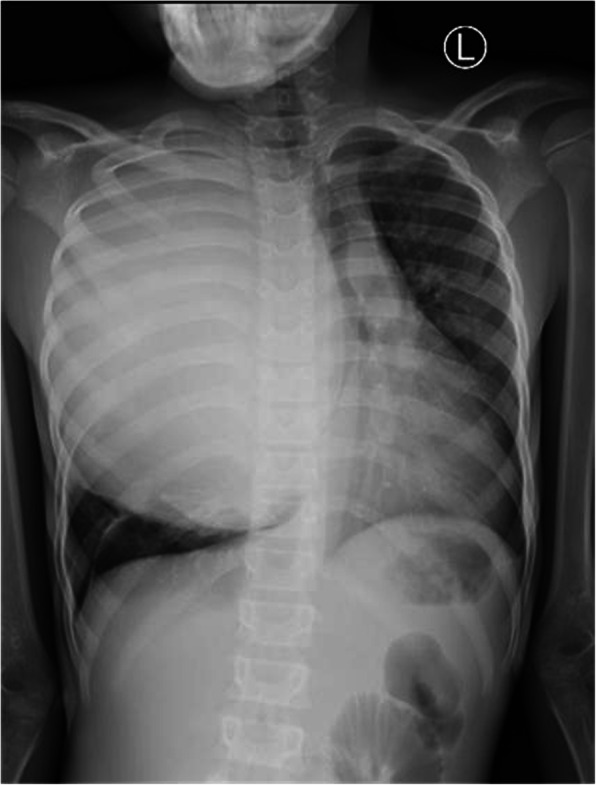
Chest x-ray posteroanterior view shows a large, rounded opacity in the right hemithorax causing mediastinal shift toward the contralateral side. Left-sided tracheal shift is seen with right main bronchus compression. No rib destruction is visualized. Features are suggestive of a hydatid cyst

**Fig. 4:**
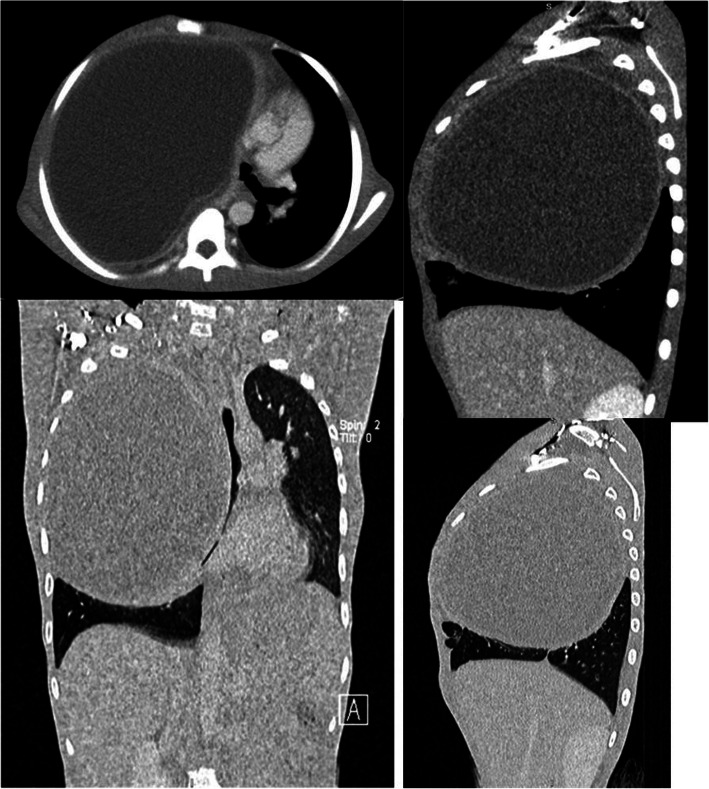
Contrast-enhanced chest computed tomography in axial, coronal, and sagittal views demonstrating a large cystic lesion in the right upper and middle hemithorax measuring 15.5 cm (AP) × 11.3 cm (T) × 14.9 cm (CC) and displacing the mediastinum to the contralateral side of the chest. The cystic lesion has a thick rind with double membranes on its anterior and posterior walls. No internal architecture or daughter cysts are seen. Features are suggestive of hydatid cyst. *AP* Antero-posterior, *T* Transverse, *CC* Craniocaudal

Chest expansion was more unilateral toward the right with reduced air entry and dull percussion note. No peripheral lymphadenopathy was appreciated. The findings of his abdominal and neurological examinations were unremarkable. A plain chest x-ray was obtained and showed a well-circumscribed cystic mass on the right hemithorax pushing the mediastinum toward the left (Fig. 3). A computed tomographic (CT) scan revealed a cystic lesion on the right upper and middle thorax measuring 15 × 11 × 14 cm, displacing the mediastinum to the left side of the chest and compressing the right middle and lower lung lobes. The lesion had a thick wall with clear fluid (Fig. 4). The finding of ultrasonography of the abdominopelvic region was unremarkable. His hemoglobin concentration was 12.4 g/dl, platelet count was 492 × 10^9^, leukocyte count was 7.39 × 10^9^/L, serum creatinine was 42 μmol/L, serum urea was 3.77 mmol/L, and ESR was elevated at 95 mm/hour. The finding of GeneXpert testing (Cepheid, Sunnyvale, CA, USA) for *Mycobacterium tuberculosis* was negative, and the boy’s serum electrolytes and bilirubin levels were within normal range. His urine test result was negative for leukocytes, nitrates, glucose, and proteins. We made a differential diagnosis of hydatid cyst of the right lung.

**Fig. 5:**
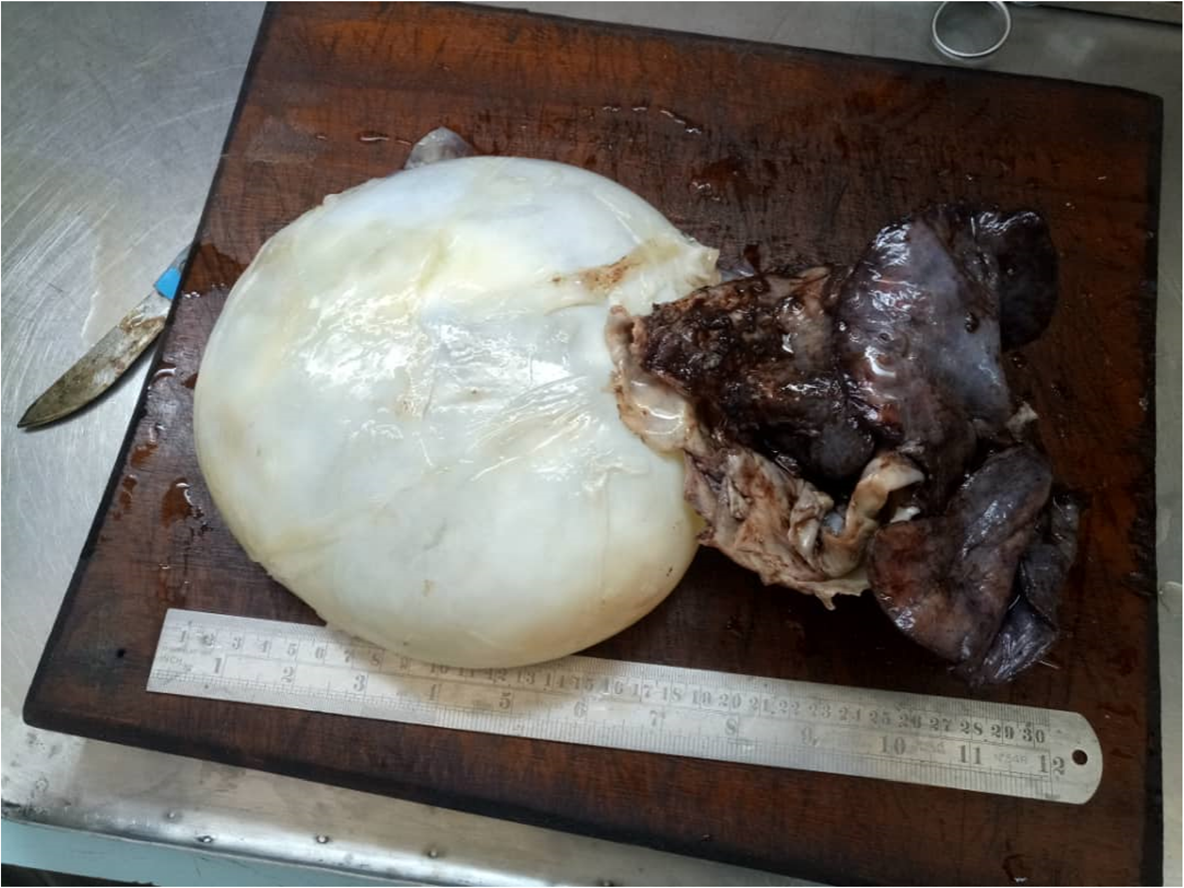
Cyst attached to lung parenchyma

**Fig. 6:**
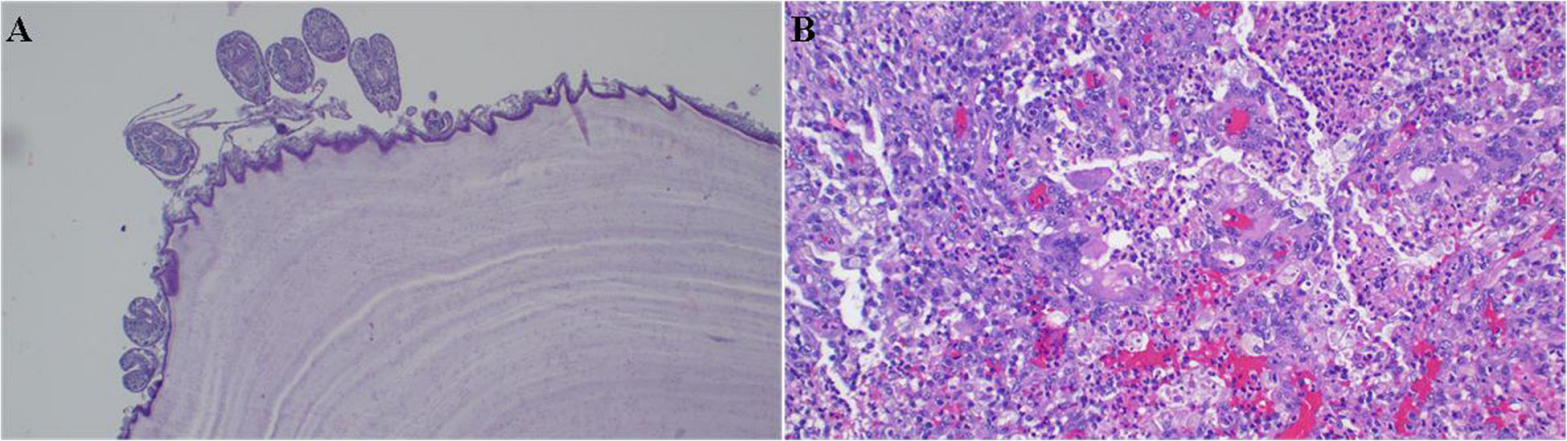
**a** The cyst comprises three layers: The outermost pericyst is fibrous; the middle ectocyst layer is laminated, hyaline, and acellular; and the inner endocyst is the germinative layer, which consists of daughter cysts and brood capsules with scolices. **b** The chronic granulomatous inflammation reaction

The child underwent right-sided thoracotomy through the eighth interspace. The large cyst was attached to the lung parenchyma and hence could not be removed; therefore, we performed a standard right pneumonectomy and sent a specimen for histopathological analysis (Fig. 5). The child was kept on intravenous paracetamol 500 mg 6-hourly, ceftriaxone 500 mg 8-hourly, and metronidazole 250 mg 8-hourly for 10 days. He was monitored in the ICU for 8 days, during which time he developed acute urine retention on day 6, and the urology team inserted a suprapubic catheter (SPC). The chest tube was removed on day 11 postoperatively. The child was then discharged on day 12 with the SPC and an instruction to attend the urology outpatient clinic after 1 month with oral albendazole 400 mg daily for 6 months. Two weeks after discharge, the child was reviewed at the surgical outpatient clinic, where he was found to be clinically stable with a healed chest wound, and the SPC was patent. The histological reports confirmed the diagnosis of hydatid cyst of the lung (Fig. 6). The child was then reviewed at the outpatient clinic 2 months later jointly with the urology team, and he was found to have no chest symptoms and a healed chest wound. His SPC was blocked, and he was voiding normally per urethra with no lower urinary symptoms.

## Discussion

This case report highlights the possible risk of serious complications, including a fatal outcome, of the surgical treatment of advanced giant isolated hydatid lung cysts, particularly in endemic areas with limited resources, contrary to most reports in the literature that have documented rather fairly good treatment outcomes.

Echinococcosis is a zoonosis caused by tapeworms of the genus *Echinococcus*. The two medically important species are *E. granulosus*, which causes cystic echinococcosis, and *E. multilocularis*, which causes alveolar echinococcosis. There are two other neotropical echinococcosis species, *E. vogeli* and *E. oligarthrus* [1, 3, 4]. They are also of public health importance due to their geographical distribution. Hydatid disease is endemic in sheep-rearing countries where sheep are in close contact with humans. Humans are infected as accidental hosts in the life cycle of the worms [2, 5].

The World Health Organization (WHO) estimates the incidence of human infection to exceed 50 cases per 100,000 person-years in echinococcosis-endemic areas. The prevalence is as high as 5–10% in parts of Argentina, China, Central Asia, and East Africa. The burden of disease is said to be a loss of 1–3 million disability-adjusted life-years annually and US $3 billion for treating cases and for loss of livestock [5]. The average postoperative death rate of cystic *Echinococcus* is 2.2%, and 6.5% of cases relapse after intervention, as stated by the WHO.

Zoonosis is common among Tanzanians due to lack of knowledge and a lifestyle with close contact with livestock because more than 75% of the population lives in rural areas [6]. Like in other sub-Saharan African countries, the burden of zoonosis in Tanzania remains high due to lack of knowledge among rural communities and inadequate diagnostic capacity, leading to misdiagnosis and hence poor management. This has led to underreporting of such diseases in the area [6].

*E. granulosus* can cause one or many hydatid cysts and mostly affects liver; lungs; and, less commonly, the bones, kidneys, spleen, and central nervous system [5]. An infected individual can go up to years without symptoms during the incubation period until the cyst is large enough to produce symptoms. About 60% of humans infected with *Echinococcus* spp. remain asymptomatic. The incubation period of alveolar echinococcosis is 5–15 years, with metastasis to the spleen and brain occurring via the hematological and lymphatic systems [4, 5]. Signs and symptoms arise when the lesion compresses adjacent structures and also depends on the locality. Both our cases presented with hemoptysis similarly to the case reported by Kant *et al.* [7]. Such cases can arise when the cyst ruptures in a bronchus, which was not present in our patients’ cases. However, when the cysts are considerably big, as in our patients’ cases, the local pressure, obstructive effect, and secondary inflammatory reaction to the adjacent bronchus may result in hemoptysis. Chest pain, breathlessness, and cough are common complaints in alveolar hydatid cysts [8]. Sarkar *et al.* also mentioned hypersensitivity reactions and anaphylaxis from the parasites or the ruptured cysts [8]. This is presumed to be the cause of death in our patient 1.

Alam *et al.* stated that in 60% the right side of the lung is affected, bilateral in 20% and 60% are located in the lower lobes. This was the case in both index patients having right-sided cysts [1]. Lodhia *et al.* previously reported a case of an isolated pulmonary hydatid cyst in a young boy that was also located on the right side of the chest and was successfully removed surgically [9]. Lungs are the second most common site for hydatid cysts, accounting for 20–30%, with the most common site being the liver at 60% [2, 8, 9]. Ramos *et al*. also mentioned a single lung cyst similar to the index cases presented, and those authors also stated that the initial period may be asymptomatic. They continued to mention rather rare but severe complications such as bronchial fistulization, intrapleural rupture, and secondary metastatic hydatidosis causing right ventricular failure, which should be taken into consideration because it makes management difficult [10].

Various investigative modalities are available for diagnosis of hydatid cysts. Numerous serological tests can be used, including enzyme-linked immunosorbent assay, indirect hemagglutination test, agglutination test, and immunoblot test, depending on availability, because humans do not discharge eggs. Radiological investigations such as plain x-rays or CT scans will provide further details of the locality of the cyst in relation to other structures and facilitate planning of surgery [5, 11]. Radiological signs such as the serpent sign, water lily sign, signet sign, or inverse crescent sign can help determine the complexity of the cyst [2, 11].

Management of lung hydatid cysts is essentially surgical, and the mortality is 1–2% with a recurrence rate of 1–3% [10, 11]. Options include lobectomy, pneumonectomy, pericystectomy, or endocystectomy, depending on the nature and extent of the cyst with regard to the lung parenchyma [11]. Both index cases underwent thoracotomy, and pneumonectomy was performed in the second case; the cyst was adherent to the lung tissues. The cyst can be infiltrated with 20% hypertonic saline if it cannot be excised, but spillage should be avoided due to the adverse effects. Rupture of the cyst can cause severe anaphylaxis; hence, the puncture, aspiration, injection, and reinjection technique can be used to avoid this complication [3, 12]. Pharmacotherapy can also be opted for when surgery is contraindicated, such as in a patient whose condition is fragile, and augmented with surgery to avoid recurrences [10]. Albendazole and mebendazole can be used. Albendazole is the drug of choice because its penetration and systemic absorption are high [1, 11]. Treatment is indicated for 3–6 months. Generally, cysts less than 5 cm can be managed nonsurgically, and those larger are drained percutaneously or surgically excised following treatment with antihelmintics [9]. Follow-up needs to be individualized because there are no formal recommendations. Generally, albendazole should be prescribed for 6 months to avoid recurrence, which is as high as 11%, and plain chest x-rays should be obtained monthly for the first 3 months [1, 13].

## Conclusion

Cystic echinococcosis remains a significant problem worldwide in terms of medical, social, and economic impacts. Surgery is the mainstay of management of pulmonary hydatidosis, and pharmacological therapy can be sought in patients with surgical contraindications. Surgery carries a high risk of rupture, especially if the cysts are considerably big due to late presentation and thus pose a risk of fatal outcome, particularly in resource-limited settings. Disease control can be improved by increasing awareness among health care workers and educating the community before more importance is given to increasing diagnostic capacity and implementing prevention and control strategies suggested by the WHO and the Centers for Disease Control and Prevention, aiming at high-risk groups such as the Maasai in countries such as Tanzania, where the majority of the population are in close contact with cattle.

## Data Availability

All data used in this study are available from the corresponding author upon request.

## Abbreviations

CT: Computed tomographic
ESR: Erythrocyte sedimentation rate
ICU: Intensive care unit
SPC: Suprapubic catheter
WHO: World Health Organization
